# Unveiling the drivers of carbon sequestration in Baotou shelterbelt species: A functional group perspective with xgboost-SHAP interpretation

**DOI:** 10.1016/j.isci.2026.115613

**Published:** 2026-04-06

**Authors:** Shumin Han, Yu Liu, Penghao Ji, Runhong Gao, Rui Zhang, Wenying Zhang, Jingtao Weng

**Affiliations:** 1 College of Forestry, Inner Mongolia Agricultural University, Inner Mongolia, Hohhot 010019, China; 2 College of Science, Inner Mongolia Agricultural University, Inner Mongolia, Hohhot 010018, China; 3 Baotou Forestry and Grassland Work Station, Inner Mongolia, Baotou 014030, China

**Keywords:** geology, applied geology, geochemistry, global carbon cycle, computer modeling

## Abstract

Understanding the drivers of carbon sequestration in semi-arid shelterbelts is crucial for precision species selection and adaptive management. This study integrated multi-seasonal photosynthetic measurements of 22 species in Baotou, Inner Mongolia, with functional group classification and an explainable XGBoost-SHAP framework. Four distinct photosynthetic functional groups were identified, with XGBoost achieving high predictive skill for net photosynthetic rate (Pn) with a test set R2 of 0.897. SHAP interpretation revealed group-specific drivers: high Pn groups were primarily limited by photosynthetically active radiation (PAR), while others exhibited stronger stomatal conductance (gs) regulation. Notably, vapor pressure deficit (VPD) triggered an inhibitory transition near 2.1 kPa and a 95% confidence interval from 1.379 to 3.93 kPa. These findings provide a threshold-informed, functional group-based strategy for precision afforestation and enhancing carbon sequestration in semi-arid systems, supporting large-scale initiatives such as the Three-North Shelterbelt Program.

## Highlights

• XGBoost-SHAP framework unveils drivers of shelterbelt carbon sequestration
• 22 species are classified into 4 functional groups based on traits
• Pn inhibition thresholds, such as 2.1 kPa VPD, are identified for carbon capture
• Explainable AI aids precision management for semi-arid forest restoration

## Graphical abstract

**Figure:**
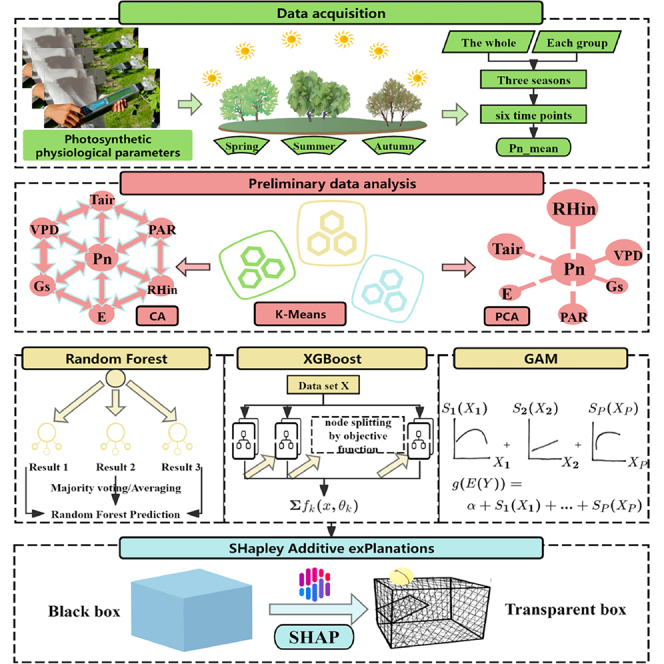

## Introduction

In the context of global climate change, enhancing the carbon sequestration of terrestrial ecosystems has become a critical pathway for mitigating rising atmospheric CO_2_ concentrations. As a core carbon pool, the precise improvement of forest carbon sequestration is of great significance for China to achieve its Dual Carbon goals.^1^^,^^2^ The Three North Shelterbelt Forest Program, the world’s largest artificial ecological project, holds not only remarkable importance in windbreaking and sand fixation but also immense carbon sequestration potential.^3^^,^^4^ This potential is particularly crucial in fragile semi-arid regions, where shelterbelts serve both as ecological barriers and key components of the regional carbon budget.^5^ Photosynthetic carbon assimilation in trees is fundamentally governed by a suite of environmental and physiological factors. In semi-arid regions, the impact of Photosynthetically active radiation (PAR) is particularly pronounced due to intense radiation and significant temperature fluctuations.^6^^,^^7^ Concurrently, vapor pressure deficit (VPD) imposes a critical atmospheric stress, directly and indirectly regulating stomatal aperture and thus the trade-off between carbon gain and water loss.^8^^,^^9^ Stomatal conductance (gs) governs the rate of water evaporation and CO_2_ uptake in plants, making it one of the key limiting factors in photosynthesis.^10^ Therefore, identifying the key environmental drivers of photosynthetic carbon sequestration in shelterbelt species within semi-arid regions is crucial for optimizing forest structure, enhancing carbon sequestration potential, and supporting the strategic shift in ecological engineering from a focus on large-scale greening to a focus on functional efficacy.^11^

Current research on plant carbon sequestration capacity primarily focuses on leaf-scale photosynthetic parameters and their responses to environmental factors.^11^^,^^12^^,^^13^ The classification of plant functional groups is widely used to interpret differences in resource use strategies among species.^14^^,^^15^ However, this field faces several pronounced limitations. Firstly, most studies are confined to single species or seasons, lacking systematic cross-seasonal, multi-species analyses at the functional group level. This hampers the extraction of universal patterns to guide species allocation at regional scales.^16^^,^^17^ Secondly, methodologically, reliance on traditional linear models, such as correlation and principal component analysis (PCA), remains prevalent.^18^^,^^19^ Ecological processes are inherently nonlinear, characterized by complex nonlinear relationships and interactions between environmental factors and photosynthesis.^20^^,^^21^ Photosynthetic limitation refers to the phenomenon where photosynthetic rates fall below their potential maximum due to factors such as gs and biochemical reaction rates. It is typically categorized into three types: stomatal limitation, mesophyll limitation, and biochemical limitation. In semi-arid regions, variations in environmental factors often affect plant carbon fixation capacity through the combined effects of gs and non-stomatal limitations.^22^ Linear models struggle to accurately capture these patterns, resulting in insufficient depth in mechanistic understanding and limited predictive capability.^23^ These constraints diminish the applied value of research findings in supporting precision silviculture for shelterbelt forests.

To overcome the bottlenecks of species-specific analyses and the limited interpretability of purely data-driven models,^24^^,^^25^^,^^26^ this study proposes an integrated framework that couples functional ecology with explainable machine learning to quantify how environmental drivers regulate photosynthesis^27^^,^^28^ and, consequently, carbon sequestration potential^29^^,^^30^ at the functional group level.^31^^,^^32^ Recent studies have increasingly adopted machine learning approaches for forest carbon estimation and, more importantly, explainable techniques to capture nonlinear relationships and attribute the contribution of key climatic, site, and biophysical factors, thereby strengthening mechanistic understanding and decision relevance.^33^^,^^34^^,^^35^ Based on multi-season photosynthetic physiological observations of 22 major shelterbelt tree species in Baotou, a typical semi-arid region of Inner Mongolia, we further test the applicability of this integrated framework under real ecosystem conditions.

Existing studies indicate that plants with contrasting combinations of functional traits differ systematically in stomatal regulation, water-use efficiency (WUE), and photosynthetic potential, which in turn leads to divergent sensitivities to environmental drivers such as light, air temperature (Tair), and VPD.^36^^,^^37^ Moreover, photosynthetic responses to environmental forcing are often markedly nonlinear. In recent years, carbon sequestration research has increasingly adopted interpretable machine learning approaches to capture complex nonlinear relationships while quantifying the relative contributions of key factors, thereby improving the interpretability of ecological inference and management applications.^34^^,^^38^ Building on these advances, we hypothesize that the carbon sequestration capacity of different photosynthetic functional groups is driven by distinct dominant environmental factors, and that these drive response relationships are mainly nonlinear. Accordingly, this study aims to: (1) establish carbon sequestration functional groups based on key photosynthetic traits; (2) elucidate the diurnal and seasonal dynamics of photosynthetic parameters across different species and functional groups; (3) identify the key environmental factors influencing each functional group through correlation and PCA; (4) systematically compare the performance of multiple machine learning models in predicting Pn; and (5) utilize SHAP analysis to quantitatively reveal the key environmental drivers and their modes of action governing carbon sequestration capacity across the entire dataset and within distinct functional groups. Our results are expected to provide quantitative evidence for carbon-sequestration-oriented species selection and precision configuration of shelterbelts in semi-arid regions.

## Results and analysis

### Divergence in photosynthetic physiological strategies and identification of carbon sequestration functional groups in shelterbelt species

**Figure 1:**
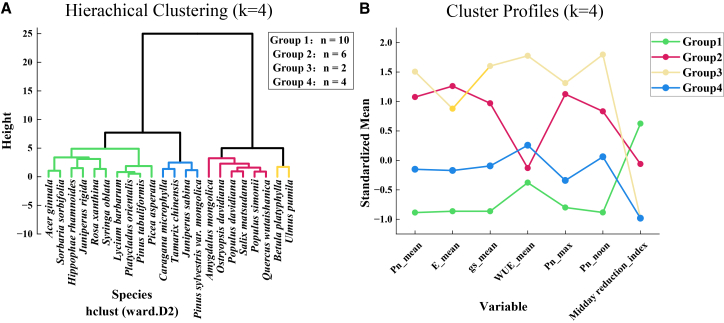
Cluster analysis results (A) Hierarchical clustering dendrogram of tree species with K = 4. (B) Cluster profiles of standardized mean of variables with K = 4.

Cluster analysis separated the 22 shelterbelt species into four photosynthetic functional groups, as shown in Figure 1. The classification relied on a trait set that captures four key dimensions of photosynthetic carbon sequestration under semi-arid conditions: carbon gain capacity, stomatal gas exchange and water loss, the carbon water trade-off, and diurnal stability. These dimensions were represented by Pn_mean, Pn_max, and Pn_noon, gs_mean and E_mean, WUE_mean, and the midday reduction index, respectively, with details reported in Table 1. One-way ANOVA indicated that the four groups differed significantly in the major physiological traits with *p* < 0.05, supporting the reliability of the functional grouping.

**Table 1:** Mean physiological and ecological parameters for each cluster

Functional Group	Pn_mean	E_mean	Gs_mean	WUE_mean	Pn_max	Pn_noon	Midday reduction _index
Group 1	2.49 ± 0.32 c	0.83 ± 0.1 c	15.24 ± 2.21 ab	3.38 ± 0.17 b	4.49 ± 0.62 ab	1.98 ± 0.27 days	0.54 ± 0.05 b
Group 2	8.19 ± 0.3 a	2.46 ± 0.09 a	56.37 ± 3.16 a	3.52 ± 0.12 ab	12.57 ± 0.75 a	7.2 ± 0.68 a	0.41 ± 0.07 ab
Group 3	9.44 ± 0.21 a	2.16 ± 0 a	70.58 ± 15.89 b	4.6 ± 0.18 a	13.36 ± 0.54 b	10.13 ± 0.87 b	0.24 ± 0.03 ab
Group 4	4.62 ± 0.29 b	1.36 ± 0.13 b	32.52 ± 3.25 b	3.74 ± 0.29 ab	6.42 ± 0.47 b	4.86 ± 0.36 c	0.24 ± 0.03 a

Data in the table are presented as mean ± standard error. Different lowercase letters indicate significant differences between groups at *p* < 0.05.

**Table 2:** Basic plants information

Life form	Family Name	Scientific Name	Mean plant height	Mean DBH	Number of Trees	Density
			H/m	DBH/cm		individuals/ha
Tree	Sapindaceae	*Acer ginnala* Maxim.	6.53 ± 0.45	15.77 ± 0.75	36	900
	Betulaceae	*Betula platyphylla* Sukaczev	6.13 ± 0.46	13.01 ± 0.55	20	500
	Cupressaceae	*Juniperus rigida* Siebold & Zucc.	4.12 ± 0.27	5.93 ± 0.24	26	650
	Pinaceae	*Picea asperata* Mast.	2.53 ± 0.36	3.09 ± 0.33	36	900
	Salicaceae	*Populus davidiana* Dode	11.84 ± 0.63	12.36 ± 0.54	43	1075
	Cupressaceae	*Platycladus orientalis* (L.) Franco	2.1 ± 0.32	3.1 ± 0.35	37	925
	Salicaceae	*Populus simonii* Carr.	13.19 ± 0.65	19.49 ± 0.42	37	925
	Pinaceae	*Pinus sylvestris* var. *mongolica* Litv.	4.88 ± 0.32	8.83 ± 0.42	35	875
	Pinaceae	*Pinus tabuliformis* Carr.	13.03 ± 0.67	30.61 ± 1.32	15	375
	Fagaceae	*Quercus wutaishanica* Mary	6.2 ± 0.43	16.13 ± 0.68	39	975
	Salicaceae	*Salix matsudana* Koidz.	6.48 ± 0.34	18.22 ± 0.74	47	1175
	Ulmaceae	*Ulmus pumila* L.	5.84 ± 0.43	10.5 ± 0.45	28	700
Shrub	Rosaceae	*Amygdalus mongolica* (Maxim.) Ricker	1.33 ± 0.17	1.41 ± 0.15	5	2000
	Fabaceae	*Caragana microphylla* Lam.	1.56 ± 0.22	1.09 ± 0.13	8	3200
	Elaeagnaceae	*Hippophae rhamnoides* L.	2.53 ± 0.24	4.12 ± 0.32	4	1600
	Cupressaceae	*Juniperus sabina* L.	1.29 ± 0.13	2.07 ± 0.22	5	2000
	Solanaceae	*Lycium barbarum* L.	0.32 ± 0.12	1.43 ± 0.07	4	1600
	Betulaceae	*Ostryopsis davidiana* Decne.	1.26 ± 0.13	2.42 ± 0.16	8	3200
	Rosaceae	*Rosa xanthina* Lindl.	1.13 ± 0.11	0.81 ± 0.04	3	1200
	Oleaceae	*Syringa oblata* Lindl.	0.14 ± 0.04	1.01 ± 0.04	6	2400
	Rosaceae	*Sorbaria sorbifolia* (L.) A. Braun	1.17 ± 0.15	2.52 ± 0.24	4	1600
	Tamaricaceae	*Tamarix chinensis* Lour.	2.36 ± 0.22	3.82 ± 0.21	3	1200

**Table 3:** Actual measurement and derived variable information

Category	Indicator	Abbrev.	Unit	Role
Measured	net photosynthetic rate	Pn	(ɥmol/m/s)	instantaneous net CO_2_ assimilation per leaf area; reflects carbon assimilation capacity.
	transpiration rate	E	(mmol/m/s)	instantaneous water vapor flux per leaf area; reflects plant water loss.
	stomatal conductance	gs	(mmol/m/s)	key trait linking CO_2_ uptake and water loss; indicates stomatal regulation strategy.
	photosynthetically active radiation	PAR	(ɥmol/m/s)	light energy input for photosynthesis.
	air temperature	Tair	(°C)	affects biochemical reaction rates related to photosynthesis.
	leaf temperature	Tleaf	(°C)	linked to enzymatic reactions and transpiration processes.
	carbon dioxide content in air	CO_2_in	(ppm)	substrate concentration for photosynthesis; affects carbon fixation efficiency.
	relative humidity of air	RHin	(%)	together with VPD, it indicates atmospheric water stress.
	vapor pressure deficit	VPD	(kPa)	key driver of stomatal behavior; indicates atmospheric dryness stress.
Derived	water use efficiency	WUE	(ɥmol/mmol)	reflecting the trade-off between plant carbon fixation and water loss.
	maximum net photosynthetic rate	Pn max	(ɥmol/m/s)	maximum Pn among all time points in the day.
	midday net photosynthetic rate	Pn noon	(ɥmol/m/s)	Pn at midday (14:00)
	midday reduction index	Midday reduction index	dimensionless	midday reduction in photosynthesis index.

The standardized trait profiles revealed clear divergence in resource use strategies across groups. Group 1, represented by species such as *Juniperus rigida* and *Pinus tabuliformis*, exhibited consistently low photosynthetic performance and the strongest midday depression, indicating a conservative strategy with limited carbon gain and low tolerance to midday stress. Group 2, including typical broadleaf species such as *Amygdalus mongolica* and *Quercus wutaishanica*, showed high Pn accompanied by high gs and E, reflecting an acquisitive strategy that achieves high carbon gain through greater stomatal openness and higher water expenditure. Group 3, consisting of *Betula platyphylla* and *Ulmus pumila*, combined exceptionally high Pn_max and Pn_noon with high gs, suggesting a high carbon assimilation potential together with a strong capacity to sustain photosynthesis under midday conditions. Group 4, represented by species such as *Pinus sylvestris* var. *mongolica* and *Juniperus sabina*, maintained moderate photosynthesis while achieving higher WUE and a low midday reduction index, indicating a more water-efficient and stress-resilient strategy for carbon gain.

Quantitatively, groups 3 and 2 showed significantly higher Pn, reaching 9.44 ± 0.21 and 8.19 ± 0.30 μmol/m^2^/s, respectively, identifying them as high photosynthesis groups. Under midday high light conditions, group 3 maintained the highest Pn_noon of 10.13 ± 0.87 μmol/m2/s, while its midday reduction index of 0.24 ± 0.03 was comparable to group 4 and markedly lower than group 1, which reached 0.54 ± 0.05. Group 3 also exhibited the highest WUE of 4.60 ± 0.18 μmol/mmol, indicating an advantageous combination of strong carbon fixation and relatively conservative water use. Overall, these results demonstrate pronounced functional differentiation in photosynthetic strategies among shelterbelt species, with groups 3 and 4 representing two superior pathways for maintaining carbon sequestration capacity under semi-arid environmental stress.

### Diurnal and seasonal dynamics of photosynthetic carbon sequestration characteristics across functional groups

**Figure 2:**
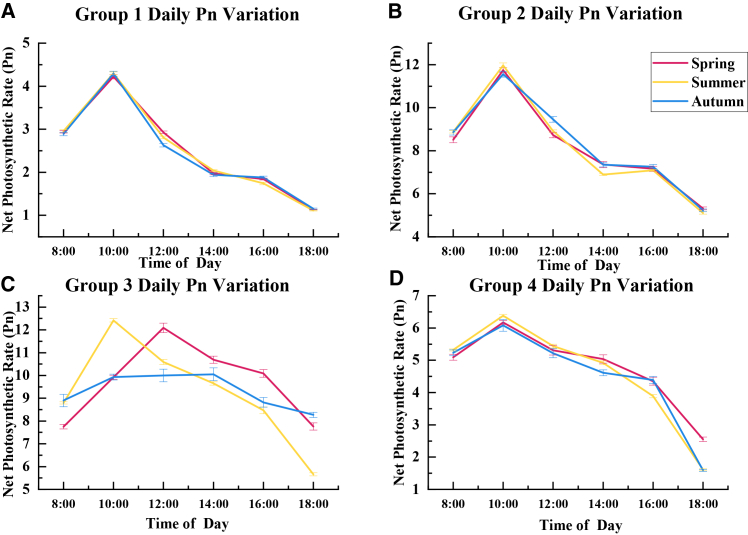
Seasonal dynamics of Pn across the four distinct functional groups (A) Pn of photosynthetic functional group 1 in spring, summer, and autumn. (B) Pn of photosynthetic functional group 2 in spring, summer, and autumn. (C) Pn of photosynthetic functional group 3 in spring, summer, and autumn. (D) Pn of photosynthetic functional group 4 in spring, summer, and autumn.

The diurnal dynamics of net photosynthetic rate, shown in Figure 2, distinguished how the four photosynthetic functional groups responded to midday stress in semi-arid shelterbelts and were consistent with the functional classifications. Group 3 exhibited the smoothest diurnal trajectory, with no obvious midday reduction when high light and temperature peaked, and Pn was sustained or even continued to increase in spring and autumn. This pattern indicates a strong capacity to maintain carbon assimilation under short-term daytime stress, which is consistent with a photoprotection and rapid recovery strategy that stabilizes carbon gain. In contrast, group 2 showed a pronounced midday reduction with a clear double-peak or V-shaped curve. Despite its high photosynthetic capacity, this pattern suggests stronger midday limitation of carbon assimilation, which is typically associated with tighter stomatal regulation under high evaporative demand and or transient biochemical constraints during peak stress. Group 4 showed a relatively stable diurnal curve with minimal midday reduction, resembling the stability pattern of group 3, but its overall photosynthetic level remained within a moderate range. This combination implies a conservative but robust strategy that maintains steady carbon gain while avoiding excessive water loss under semi-arid conditions. Group 1 displayed both low photosynthetic capacity and high instability, with the lowest Pn throughout the day and marked fluctuations. The unstable and depressed diurnal profile suggests lower physiological resilience to rapid environmental changes, which is consistent with higher sensitivity of photosynthetic processes to midday stress.

**Figure 3:**
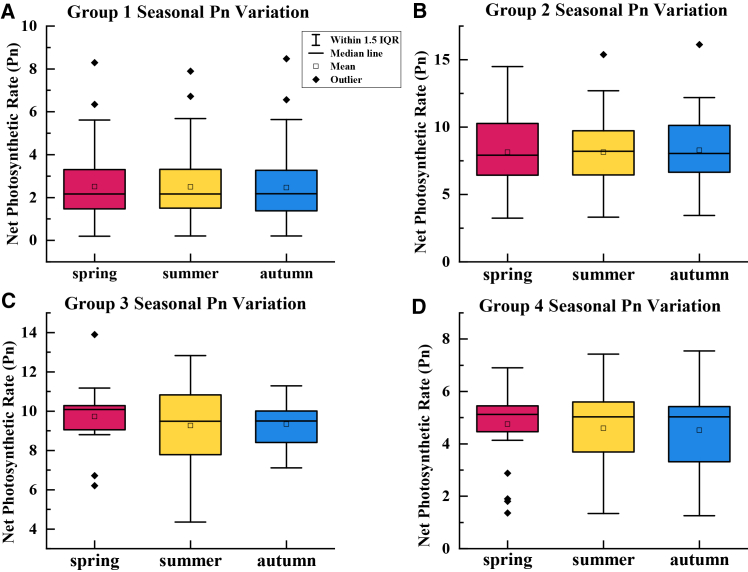
Statistical distribution of seasonal variations in Pn for each functional group (A) Boxplots of seasonal variation in Pn for photosynthetic functional group 1. (B) Boxplots of seasonal variation in Pn for photosynthetic functional group 2. (C) Boxplots of seasonal variation in Pn for photosynthetic functional group 3. (D) Boxplots of seasonal variation in Pn for photosynthetic functional group 4.

Seasonal boxplots of Pn, shown in Figure 3, further revealed group-level differences in stability across spring, summer, and autumn. Groups 2 and 3 maintained relatively high median Pn with limited dispersion across seasons, indicating consistently higher carbon assimilation capacity. Group 3 retained strong photosynthetic performance during summer heat and drought, suggesting higher tolerance to seasonal intensification of water and thermal stress. Group 4 showed the most stable seasonal pattern, with minimal variation in median Pn and interquartile range, reflecting a steady strategy that supports reliable carbon gain under fluctuating environments. In contrast, group 1 remained low in Pn across seasons and exhibited higher within-group variability, indicating weaker and more heterogeneous responses to seasonal stress. Together, the diurnal and seasonal patterns highlight clear temporal differentiation in carbon assimilation among functional groups, providing physiological evidence for functional-group-based species configuration in semi-arid shelterbelt systems.

### Relationships between environmental factors and photosynthetic carbon sequestration capacity, and the dominant environmental patterns

**Figure 4:**
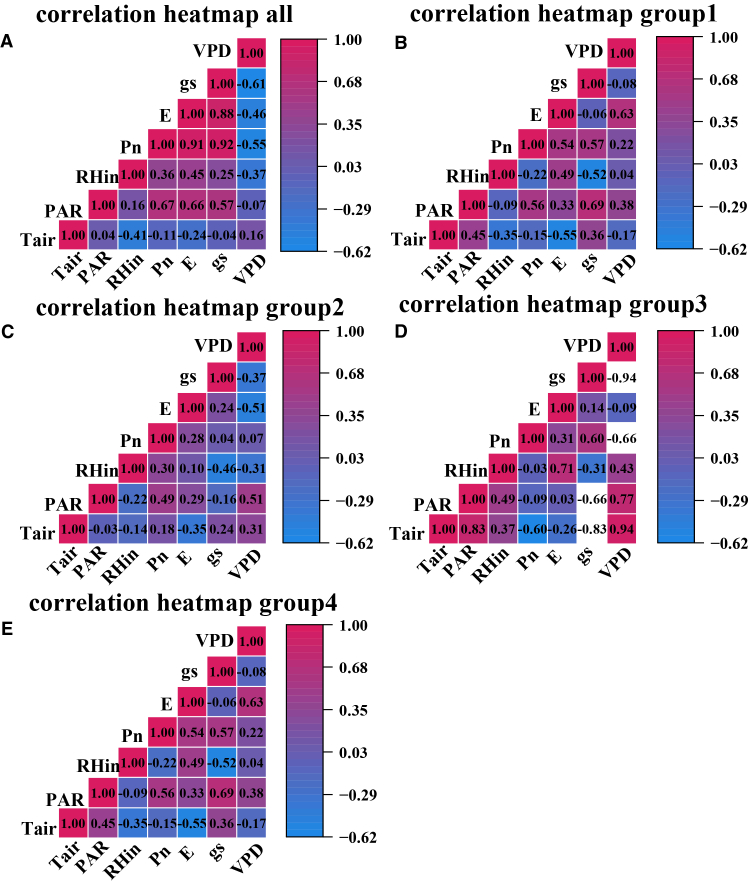
Spearman’s correlation analysis between Pn and environmental factors (A) Spearman’s correlation heatmaps between Pn and environmental factors in photosynthetic functional all data groups. (B) Spearman’s correlation heatmaps between Pn and environmental factors in photosynthetic functional group 1. (C) Spearman’s correlation heatmaps between Pn and environmental factors in photosynthetic functional group 2. (D) Spearman’s correlation heatmaps between Pn and environmental factors in photosynthetic functional group 3. (E) Spearman’s correlation heatmaps between Pn and environmental factors in photosynthetic functional group 4.

Spearman correlation patterns showed that the environmental regulation of net photosynthesis differed among functional groups rather than following a single shared rule. Correlation analysis revealed a fundamental shift in stomatal regulatory strategies, shown in Figure 4. The drought-adapted tree species maintained relatively high gs and transpiration under increasing irradiance, as indicated by the strong positive correlations of Pn with gs and E and the negative correlation with VPD. Together with our evidence that water stress in these species is dominated by non-stomatal limitations, this physiological behavior reduces the extent of midday depression and results in an overall strong positive relationship between Pn and PAR. The typical pattern observed across all data groups, in which Pn was tightly coupled with gs and E with a correlation coefficient ρ greater than 0.65 and was significantly negatively correlated with VPD, a correlation coefficient ρ equal to −0.55, was profoundly altered at the functional group level. In group 1, Pn shows only modest positive correlations with PAR and gs, indicating that no single external driver dominates. The absence of any strong correlation with Pn suggests that photosynthesis in group 2 is not predominantly governed by a single factor, pointing to the potential dominance of non-stomatal limitations or complex interactive effects. In group 3, Pn exhibited a stronger negative response to VPD than its positive association with gs. This pattern suggests that non-stomatal limitations play a more critical role in regulating photosynthesis under these conditions. In group 4, Pn again correlates positively with gs; instead, the Pn with VPD correlation is weak and slightly positive, implying that this group does not follow a strongly conservative, VPD avoidance strategy but tends to maintain photosynthesis even under relatively dry air conditions.

**Figure 5:**
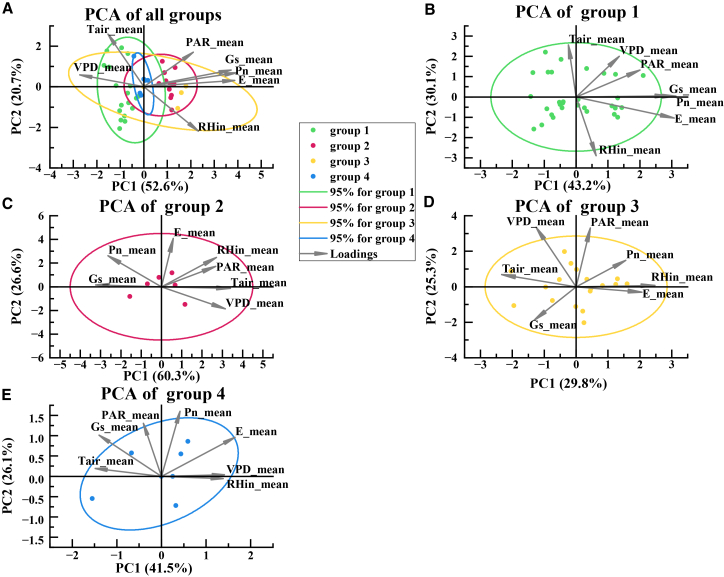
PCA of environmental factors influencing the photosynthetic functional groups (A) PCA biplots based on environmental factors in photosynthetic functional groups. (B) PCA biplots based on environmental factors in photosynthetic functional group 1. (C) PCA biplots based on environmental factors in photosynthetic functional group 2. (D) PCA biplots based on environmental factors in photosynthetic functional group 3. (E) PCA biplots based on environmental factors in photosynthetic functional group 4.

PCA further summarized how each functional group occupied a distinct region of the joint environmental and physiological space, shown in Figure 5. For the pooled dataset, the first two principal components explained 73.3% of the total variance. Group-specific PCA revealed clear differences in dimensionality. Group 3 showed the strongest compression, with the first two components explaining 86.9% of the variance, indicating that its observations aligned along a relatively consistent dominant gradient, consistent with its strong VPD sensitivity in the correlation patterns. In contrast, group 2 showed the weakest compression, with the first two components explaining 55.1% of the variance, indicating that its variability was distributed across multiple independent axes, consistent with the weak pairwise correlations and the likelihood of interactive controls. Groups 1 and 4 were intermediate, with the first two components explaining 73.3% and 67.6% of the variance, respectively. The PCA centroids also differed among groups along the first axis, with group 1 shifted to negative values, Groups 2 and 3 shifted strongly positive, and group 4 located near the origin, reflecting systematic differences in the combinations of irradiance, humidity, and evaporative demand experienced and tolerated by each group. Together, these results provide a physiological explanation for the functional classification and clarify why the diurnal and seasonal dynamics diverge among groups under semi-arid conditions.

In summary, the findings of this section serve as a crucial bridge connecting phenomena and mechanistic interpretation, fulfilling a dual role. They not only provide solid mechanistic support for the functional group classification in Section 3.1 and the photosynthetic dynamics in Section 3.2 from an environmental driving perspective, but, more importantly, they clearly reveal the limitations of traditional statistical methods: Correlations cannot capture complex nonlinear relationships, and PCA can identify patterns but cannot quantify specific contributions. These limitations provide a compelling rationale for introducing the XGBoost-SHAP approach to explainable machine learning in the next section. This model will move beyond the preliminary qualitative judgments made here and allow precise quantification and attribution of the regulatory mechanisms identified above, thereby fully addressing the deeper scientific questions of to what extent each environmental factor contributes and how nonlinearly it does so.

### Performance comparison and selection of machine learning models for predicting Pn

**Figure 6:**
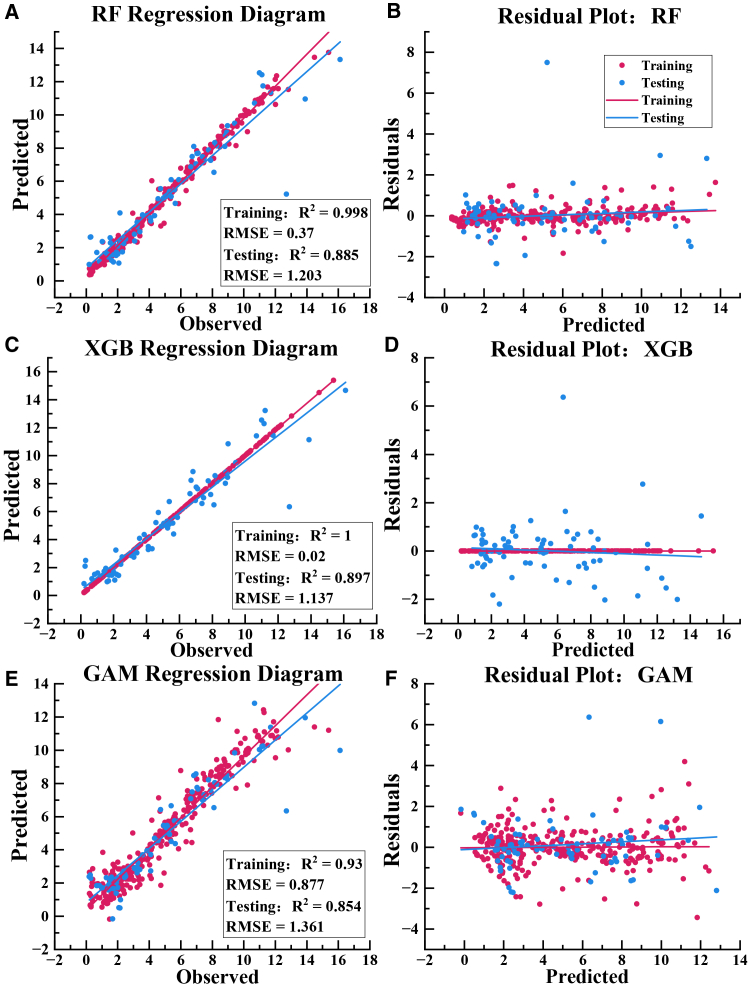
Comparison of predictive performance for Pn among three machine learning models (A and B) Prediction regression plot and residual plot for the RF model. (C and D) Prediction regression plot and residual plot for the XGBoost model. (E and F) Prediction regression plot and residual plot for the GAM.

Three regression models, RF, XGBoost, and GAM, were evaluated for predicting Pn, shown in Figure 6. The models were trained using Pn as the response variable and synchronously measured environmental factors as predictors. The full dataset was randomly partitioned into a training subset accounting for 70% of the observations and a testing subset accounting for 30%. Under the same data partition and tuning strategy, XGBoost achieved the best predictive performance on the test set, with the highest explained variance, R^2^ of 0.897, and the lowest prediction error, RMSE of 1.137. Its MAE was also the lowest among the three models, indicating consistently reduced absolute deviation and supporting the selection of XGBoost as the primary model for subsequent mechanism analysis.

Diagnostic plots further supported the reliability of XGBoost. Predicted values closely aligned with observed values along the 1:1 reference line, while residuals exhibited a random and homogeneous distribution without systematic bias or evident heteroscedasticity. These findings collectively indicate that XGBoost successfully captured the underlying dynamics of Pn variation and possessed excellent generalization ability, rather than merely memorizing the training data. Its robustness was further corroborated by 10-fold cross-validation, which yielded a high mean test set R^2^ of 0.928 with minimal fluctuation.

Performance trade-offs among the models highlighted inherent algorithmic differences. The RF model demonstrated predictive capability comparable to XGBoost, with a test set R^2^ of 0.885 and RMSE of 1.203, while exhibiting significantly higher training efficiency due to the parallelizable nature of its bagging ensemble strategy. However, XGBoost, through its boosting mechanism, achieved a marginal gain in predictive accuracy at a higher computational cost, with a training time of 100.1 s compared with 15.3 s for RF. The near-perfect fit of XGBoost on the training set, with R^2^ approaching 1, clearly signals a risk of overfitting and the incorporation of data noise. Although the regularization terms embedded in the algorithm currently alleviate this problem and sustain its leading performance on the test set, the presence of overfitting must be explicitly considered in the next stage of model development to identify ways to optimize the model and minimize overfitting. In contrast, the GAM model, while offering inherent interpretability and the fastest training speed, showed relatively lower predictive performance on the test set, with an R^2^ of 0.854. In conclusion, the model selection in this study was based on a multi-criteria framework. XGBoost was chosen as the optimal model due to its combined strengths in handling nonlinear relationships, feature interactions, and overfitting control, without compromising predictive accuracy and robustness. This decision establishes a reliable computational foundation for the subsequent application of SHAP analysis, enabling high fidelity and interpretable dissection of the driving mechanisms.

### Unveiling the drivers of carbon sequestration in shelterbelt species using XGBoost-SHAP analysis

**Figure 7:**
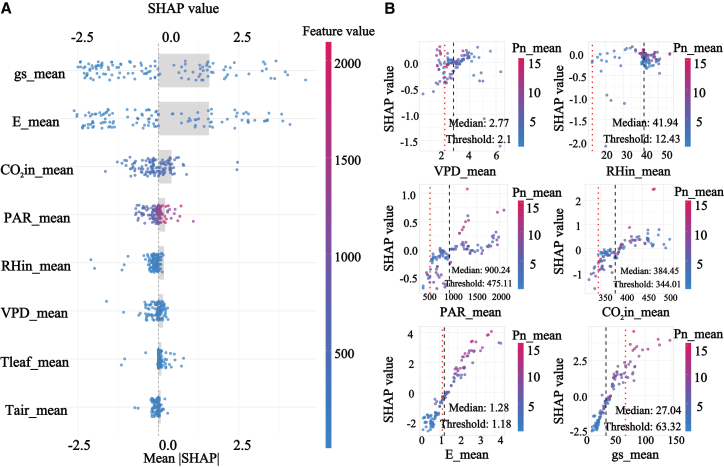
SHAP analysis of Pn across all data groups (A) presents the feature importance summary plot ranked by mean absolute SHAP value. (B) presents the SHAP dependence plots for the key environmental factors.

SHAP results based on the selected XGBoost model showed that gs and E ranked as the most influential predictors at the overall scale, indicating that gas exchange processes dominate the physiological regulation of Pn across shelterbelt species, shown in Figure 7. A clear threshold pattern was detected for VPD, with an evident turning point around 2.1 kPa and a 95% confidence interval from 1.379 to 3.93 kPa. Below this threshold, the SHAP contribution of VPD was close to zero, whereas above it, VPD exerted a strong negative contribution. This pattern is consistent with semiarid physiology in which increasing atmospheric dryness intensifies stomatal limitation and can propagate toward downstream biochemical constraints, thereby providing a quantitative early warning reference for aridity stress in shelterbelt management.

**Figure 8:**
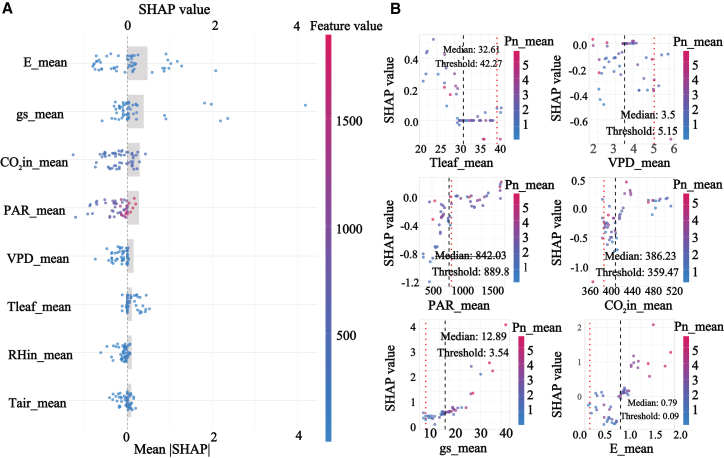
SHAP analysis of Pn in group 1 (A) presents the feature importance summary plot ranked by mean absolute SHAP value. (B) presents the SHAP dependence plots for the key environmental factors.

**Figure 9:**
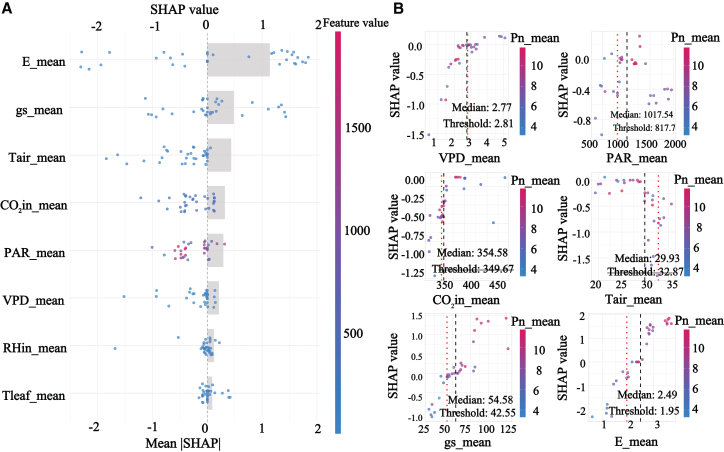
SHAP analysis of Pn in group 2 (A) presents the feature importance summary plot ranked by mean absolute SHAP value. (B) presents the SHAP dependence plots for the key environmental factors.

**Figure 10:**
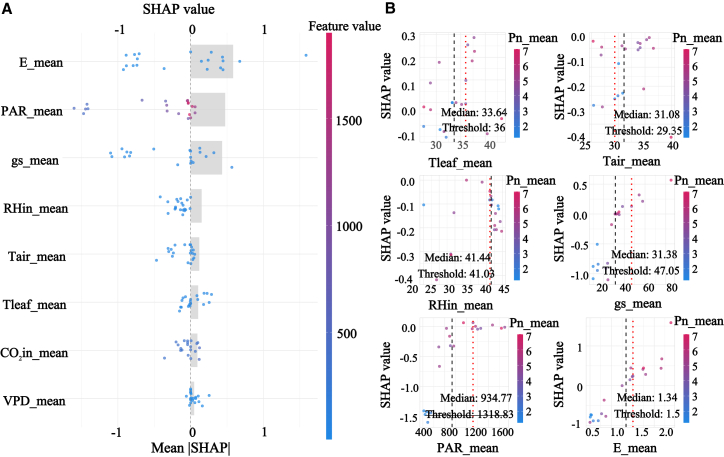
SHAP analysis of Pn in group 4 (A) presents the feature importance summary plot ranked by mean absolute SHAP value. (B) presents the SHAP dependence plots for the key environmental factors.

**Figure 11:**
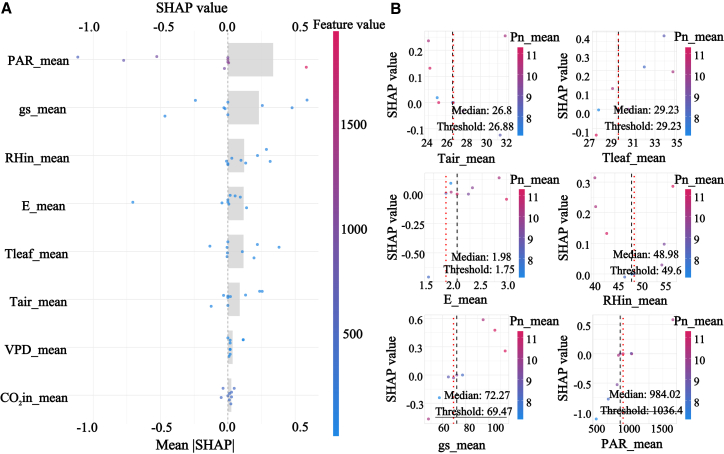
SHAP analysis of Pn in group 3 (A) presents the feature importance summary plot ranked by mean absolute SHAP value. (B) presents the SHAP dependence plots for the key environmental factors.

Functional group-specific analyses revealed distinct driving modes that aligned with observed diurnal and seasonal dynamics. Group 1, characterized by low photosynthesis and strong midday photoinhibition, was jointly regulated by gs, E, CO2in, and PAR, and the relatively low test performance with R2 of 0.689 suggests high sensitivity to fluctuating conditions and compounded limitations, shown in Figure 8. Group 2 exhibited a more focused mode dominated by E, gs, and Tair, supporting a high carbon gain strategy reliant on transpirational cooling and stomatal regulation, which is consistent with its tendency toward midday depression under peak stress, shown in Figure 9. Group 4 showed the most robust and predictable response with the highest test accuracy, R2 of 0.888, and was governed by E, PAR, and gs, indicating broader environmental adaptability and more stable seasonal behavior, shown in Figure 10. The most striking outcome emerged for group 3. In this group, PAR replaced gas exchange variables as the primary driver of Pn, and its SHAP contribution remained consistently positive across a wide irradiance range from 500 to 1500 μmol/m2/s, matching the functional definition of midday photoinhibition tolerance, shown in Figure 11. Meanwhile, the contribution of gs was markedly reduced, and its SHAP values showed a weak association with Pn. Together, these results suggest that photosynthesis in group 3 is predominantly governed by nonstomatal limitations, potentially through sustained photochemical capacity and enhanced photoprotection, enabling high carbon fixation while reducing sensitivity to stomatal restriction under high VPD. This mechanism also explains the flatter diurnal trajectories and stronger performance under summer stress reported for this functional group.

## Discussion

### Establishing a generalizable framework for ecological restoration assessment

A central challenge in ecological restoration lies in upscaling plot-level ecophysiological observations into quantifiable decision support systems capable of guiding regional management. Traditional approaches, reliant on limited field trials and expert opinion, often prove inadequate in quantifying the complex, non-linear interactions between tree species and their environment.^39^^,^^40^ This limitation was evident in our preliminary analyses. Traditional correlation analysis and PCA successfully revealed distinct environmental driving strategies among the different photosynthetic functional groups, including the marked decoupling of photosynthesis from gs in group 3 and their clear niche separation in the PCA ordination space. However, these linear and descriptive methods fell short of quantifying the specific contribution of individual environmental factors and failed to capture critical non-linear relationships, such as the inhibitory threshold of VPD on photosynthesis. This methodological gap underscores a significant bottleneck in advancing ecological restoration research from phenomenological description to mechanistic interpretation.^41^

The results demonstrate that linking functional group classification with predictive modeling and SHAP-based explanation enables a more coherent interpretation of species-level heterogeneity in Pn and its environmental drivers, thereby reducing the interpretive gap left by conventional single-method analyses. Unlike previous studies that primarily employed machine learning to simulate ecosystem patterns,^42^^,^^43^ the innovation of our framework lies not in predictive accuracy per se, but in its capacity to translate superior predictive performance into a clear ecological mechanism. First, the *a priori* classification of functional groups injects robust ecological prior knowledge into the model. This avoids the obscurity that arises from treating all species as a homogeneous population,^44^ enabling the subsequent machine learning model to more precisely learn the distinct physiological response patterns of each functional group. The exceptional performance of the XGBoost model not only validates the reliability of the predictions but also corroborates, from the results, that functional groups serve as an efficient bridge connecting micro-physiology to macro-performance.

SHAP-based response patterns and contribution estimates provide interpretable, model-grounded evidence that helps translate predictive outputs into physiologically informed and testable mechanistic hypotheses, particularly by revealing nonlinear responses and group-specific driver differences that are not readily resolved by traditional approaches. It serves as a transparent decision dashboard for an otherwise high-performance predictive black box. It quantitatively confirmed the non-stomatal limitation hypothesis in group 3, initially suggested by correlation analysis, and precisely measured the contribution of factors such as PAR; moreover, it transcended the spatial description of PCA by identifying the key VPD threshold at 2.1 kPa and a 95% confidence interval from 1.379 to 3.93 kPa. This depth of explainability resonates with the concept of machine learning-assisted planning,^45^ but our study takes a step further by implementing this concept into a standardized workflow that delivers actionable outputs, such as specific environmental thresholds and differentiated driving modes.

The generalizability of our framework is manifested in its standardized workflow, explainable outputs, and direct decision support capability. Inputting species functional traits and environmental data for a specific region allows the framework to automatically generate a functional group suitability map and key environmental factor management thresholds for that area. This provides a powerful and versatile tool for major ecological projects, such as the Three North Shelterbelt Program. It supports a shift from uniform, one-size-fits-all planning to species selection tailored to local site conditions, and from qualitative, experience-based decisions to quantitatively informed design for precision ecological restoration. Future work should integrate remote sensing and climate change scenario modeling to achieve the leap from point-based assessment to regional prediction, thereby enhancing the spatial applicability of the assessment.^46^^,^^47^

### Reconciling high photosynthesis and high WUE: Challenging the carbon-water trade-off through non-stomatal limitations

At the functional group level, the results show distinct driver structures among contrasting strategies. In particular, the high efficiency group with greater tolerance to midday stress displays an apparent decoupling between Pn and gs, and model-based attribution suggests a stronger contribution of PAR than gs to predicting Pn. This pattern provides evidence-based indications that nonstomatal processes may play an important role in sustaining photosynthesis under specific conditions, and it opens up distinct interpretive space for reassessing carbon water relationships in semiarid ecosystems as potentially governed by multiple regulatory pathways.

The classical carbon-water trade-off paradigm posits that plants cannot simultaneously maximize carbon assimilation and WUE, because greater photosynthetic CO_2_ uptake generally requires increased stomatal openness and thus incurs disproportionately high transpirational water loss.^48^^,^^49^ In this study, group 3, characterized by both high Pn and elevated WUE, appears to deviate from this canonical trade-off, thereby challenging the conventional expectation of a strict coupling between carbon gain and water expenditure. This apparent paradox is fundamentally rooted in gs, which jointly governs CO_2_ diffusion into the leaf and water vapor efflux. Classical photosynthesis models, such as the widely used Ball-Berry model, generally posit gs as the key limiting factor for CO_2_ entry into leaves, thereby placing stomatal behavior at the core of the photosynthetic response to the environment.^50^^,^^51^^,^^52^ The inception of this paradigm-shifting insight originated from a clear clue provided by traditional statistical methods. Correlation analysis first revealed a critical anomaly: The association between Pn and gs or E in group 3 was weak and statistically non-significant. This pronounced decoupling pattern between Pn and gs is not fully consistent with the tight coupling emphasized by the classical carbon water trade-off paradigm, and it raises the possibility that the underlying physiological basis may involve processes beyond stomatal regulation alone, including nonstomatal limitations. Importantly, group 3 comprises only two species, *Betula platyphylla* and *Ulmus pumila*. Therefore, inferences regarding the distinctive physiology of this group should be regarded as preliminary and hypothesis-generating, and their generality across a broader range of tree species requires further verification.

Subsequently, the SHAP analysis provided quantitative, model-based support for our proposed nonstomatal explanation, but it should be interpreted as an explanation of the predictive structure rather than a direct demonstration of a causal mechanism. Specifically, within the XGBoost model, PAR exhibited the largest contribution to predicting Pn, whereas the contribution of gs was comparatively smaller, indicating that under the present data and modeling assumptions, the model relied more strongly on light-related information to account for variation in Pn. Considered together with the observed decoupling between Pn and gs, this pattern is consistent with the possibility that the ability of this group to sustain relatively high Pn under midday high VPD conditions may relate in part to light-driven carbon balance and to constraints operating beyond stomatal aperture alone.^53^^,^^54^ At the same time, SHAP patterns primarily reflect the relative importance and response shape of predictors for model predictions, and they do not, by themselves, establish causal direction or regulatory pathways. Accordingly, we phrase this result as cautious support for a potential relative shift toward greater nonstomatal limitation, and we emphasize that its physiological and ecological implications motivate further validation using more direct physiological measurements and, where feasible, manipulative experiments.^55^^,^^56^

Ecologically, this mechanism represents a highly specialized water-use strategy: the synergistic achievement of high carbon sequestration and high WUE by circumventing stomatal limitations, which undoubtedly constitutes the essence of their evolutionary adaptation to semi-arid regions.^57^ In summary, progressing from statistical clues to mechanistic interpretation, this study highlights the central role of the carbohydrate trade-off paradox in shaping photosynthetic regulation in high efficiency shelterbelt species. Rather than attributing photosynthetic control solely to stomatal behavior, our results emphasize carbon balance and carbohydrate allocation as the primary framework within which stomatal and non-stomatal processes are embedded. This perspective not only refines the traditional understanding of photosynthetic limitation mechanisms in woody plants of semi-arid regions but also suggests up distinct directions for forest tree genetic improvement, prioritizing traits related to carbon balance and carbohydrate dynamics beyond classical stomatal traits.^58^^,^^59^

### From leaf physiology to stand-level carbon sequestration: Scaling from species to canopy and ecosystem

This study reveals pronounced nonlinear responses of Pn to environmental drivers in semiarid shelterbelt systems and demonstrates clear heterogeneity across functional groups. At the overall scale, Pn is most strongly associated with gas exchange-related variables, and VPD exhibits a threshold-like response. Model results indicate an evident transition near approximately 2.1 kPa and a 95% confidence interval from 1.379 to 3.93 kPa, above which the negative influence of atmospheric dryness on Pn becomes stronger, suggesting that increasing aridity can rapidly intensify photosynthetic suppression and elevate risks to carbon assimilation.

Leaf-level findings indicate that some high-efficiency shelterbelt species maintain relatively high Pn under midday elevated VPD, and model-based attribution suggests that variation in Pn aligns more strongly with PAR than with gs. This pattern highlights the potential importance of light-related carbon gain processes in sustaining midday photosynthesis under atmospheric dryness. Interpreted within the framework of the carbohydrate trade-off paradox, these patterns suggest that carbon balance and source sequestration coordination may play an important role in regulating leaf photosynthesis, whereas stomatal behavior may not be the sole or necessarily the primary determinant.^60^^,^^61^ A critical question, therefore, is the extent to which such leaf-level regulatory processes scale from individual species to the canopy and, ultimately, to ecosystem-level carbon sequestration in shelterbelt systems.

At the stand level, the expression of the carbohydrate trade-off paradox is mediated by species composition, functional group strategies, and canopy structure.^62^^,^^63^^,^^64^ Under this framework, periods of high PAR and high VPD, which would traditionally be viewed as predominantly suppressive because of stomatal closure, can still contribute substantially to daily and seasonal carbon gain when high E functional groups remain active. High E functional groups that can sustain strong light-driven carbon gain under high VPD conditions contribute disproportionately to canopy photosynthesis in exposed, well-lit crown positions, whereas more conservative groups maintain carbon uptake under less favorable microclimates.^65^^,^^66^ In this way, leaf-level carbon balance mechanisms captured by the carbohydrate trade-off paradox are amplified and integrated through canopy architecture and species complementarity to shape stand-scale photosynthetic capacity.^67^ Scaling from instantaneous leaf gas exchange to stand-level carbon sequestration therefore requires integrating these spatial patterns of canopy photosynthesis across time. Consequently, the translation of leaf photosynthesis into annual net primary production and net ecosystem carbon balance in shelterbelt forests is governed not only by the magnitude of canopy photosynthesis but also by the temporal dynamics of carbon allocation, both of which are shaped by the underlying carbohydrate trade-off relationships.^68^

These scaling relationships have direct implications for the design and management of shelterbelt systems in semi-arid regions. By deliberately combining functional groups with contrasting expressions of the carbohydrate trade-off paradox, shelterbelt stands can be configured to exploit high light, high VPD windows for carbon gain while maintaining stability under suboptimal conditions. Modular layout and VPD threshold based management provide practical levers to match species composition and canopy structure to local microclimatic regimes, thereby enhancing both stand level carbon sequestration and the broader ecosystem functions of shelterbelts.^69^ From this perspective, optimizing shelterbelt systems involves not only increasing vegetation coverage, but strategically organizing species, canopy architecture, and environmental thresholds in a way that maximizes long-term ecosystem carbon storage.

### Toward carbon neutral shelterbelt design: From mechanistic understanding to precision management

These findings support actionable implications for shelterbelt functional optimization, namely using functional group configuration to identify key environmental thresholds and sensitive periods, and translating threshold information into early warning and precision-oriented management to improve the efficiency and stability of carbon sequestration. We note that some functional groups are represented by limited species, and the present evidence is derived primarily from statistical relationships and explainable machine learning models. Future work should broaden species and site coverage and incorporate more direct physiological measurements and, where feasible, manipulative experiments to test the nonstomatal limitation hypothesis and to evaluate how leaf-level mechanisms scale to canopy and ecosystem-level carbon dynamics.

The ultimate goal of all the mechanistic insights gained in this study is to facilitate a strategic transition in shelterbelt system development from merely pursuing vegetation coverage to proactively optimizing ecosystem functions by providing a precise design blueprint and management toolkit. Based on the findings above, we propose the core concept of modular functional group configuration, which aims to maximize carbon sequestration and ecosystem stability through the targeted assembly of functional groups.^70^

The primary principle of this design is the spatial matching of sites with the inherent strategies of different functional groups. For group 3, which exhibits the synergistic advantages of high Pn and high WUE, it should be deployed in areas with relatively superior hydrothermal conditions to form core Carbon Sefficiency Hotspots, thereby maximizing regional carbon uptake. Conversely, for group 4, which employs a robust conservative strategy and exhibits high sensitivity to atmospheric drought, it should be assigned to arid and barren sites to constitute Stable Stress-Resilience Zones, leveraging its exceptional persistence to ensure long-term ecosystem resilience and service continuity.^71^ This modular design, grounded in functional traits, avoids a one-size-fits-all species selection and enhances the structural and functional stability of the entire stand through niche complementarity.^72^^,^^73^ Secondly, a key translational output of this research is the conversion of the VPD threshold with 2.1 kPa and a 95% confidence interval from 1.379 to 3.93 kPa, identified via SHAP analysis, into an actionable precision management indicator.^74^^,^^75^^,^^76^ This threshold defines the critical point at which atmospheric drought universally inhibits photosynthesis in the shelterbelt. When real-time monitoring indicates that VPD exceeds this value, it signals that trees are undergoing significant atmospheric water stress. Implementing precise supplemental irrigation for high-value trees in the Carbon Sefficiency Hotspots at this juncture can effectively mitigate photosynthetic inhibition and protect carbon sequestration capacity. This physiological threshold-based alert management represents a leap from extensive, experience-based water allocation to precision efficiency, significantly enhancing WUE and carbon sequestration gains.

In summary, these findings support a practical strategy for shelterbelt functional optimization in which functional group configuration is used to identify key environmental thresholds and sensitive periods, thereby informing targeted management and species mixture design to enhance the stability of carbon assimilation under increasing atmospheric aridity. This framework seamlessly couples deep ecological mechanisms such as functional group strategies and the carbohydrate trade-off paradox with executable engineering design, including modular layout and VPD threshold management.^77^^,^^78^ This paradigm not only provides a direct technical pathway for enhancing the quality and efficiency of ongoing projects such as the Three North Shelterbelt Program but, more importantly, demonstrates a viable route for translating ecological knowledge into a powerful tool for addressing climate change and supporting the Carbon Neutrality strategy, offering a complete chain solution from theory to practice for global ecological restoration in semi-arid regions.

### Limitations of the study

While this study identifies key drivers of carbon sequestration using an explainable AI framework, several limitations should be noted. First, the functional grouping, particularly group 3, is represented by a limited number of species (*Betula platyphylla* and *Ulmus pumila*), which may constrain the generalizability of the specific non-stomatal mechanisms identified. Second, our observations are based on leaf-level gas exchange; scaling these findings to canopy-level and ecosystem-level carbon budgets requires integration with sap flow measurements and remote sensing data. Finally, the study was conducted in a single semi-arid region, and the identified environmental thresholds (e.g., VPD at 2.1 kPa) may vary across different climatic zones.

## Resource availability

### Lead contact

Further information and requests for resources should be directed to and will be fulfilled by the lead contact, Runhong Gao (grhzwdm@163.com).

### Materials availability

This study did not generate new unique reagents or biological materials.

### Data and code availability

• Data: The original observational and analytical data reported in this paper have been deposited at Zenodo and are publicly available as of the date of publication. The DOI is 10.5281/zenodo.18931054.
• Code: All original code utilized in this study has been deposited at Zenodo and is publicly available as of the date of publication. The DOI is 10.5281/zenodo.18931054.
• Other items: Any additional information required to reanalyze the data reported in this paper is available from the lead contact upon request.

## Acknowledgments

We sincerely thank the staff at the Baotou Forestry and Grassland Work Station for their assistance with field sampling and site access. We also thank the anonymous reviewers for their constructive comments that improved this manuscript.

## Author contributions

The contributions of S.H., Y.L., P.J., R.Z., and J.W. to this manuscript are data acquisition and analysis. S.H., Y.L., R.G., and W.Z. contributed to the manuscript by writing, revising, and proofreading it. Each author agrees to take personal responsibility for their contribution.

## Declaration of interests

The authors declare no competing interests.

## STAR★Methods

### Key resources table

**Table:** 

REAGENT or RESOURCE	SOURCE	IDENTIFIER
**Deposited data**		

Original observational data and analysis results	This paper; Zenodo	https://doi.org/10.5281/zenodo.18931054

**Experimental models: Organisms/strains**		

12 kinds of trees and 10 kinds of shrubs	Local collection (Baotou, Inner Mongolia)	N/A

**Software and algorithms**		

XGBoost algorithm	Chen and Guestrin, 2016	https://xgboost.readthedocs.io/
SHAP Explanation Framework	Lundberg et al., 2020	https://github.com/slundberg/shap
R language (4.5.0)	R Core Team	RRID: SCR_001905
Zotero document management software		https://www.zotero.org/
Original analysis code	This paper; Zenodo	https://zenodo.org/records/18931054

**Other**		

CI-340 Portable Photosynthesis Measurement System	CID Bio-Science	https://cid-inc.com/

### Experimental model and study participant details

#### Field site and plant materials

**Figure 12:**
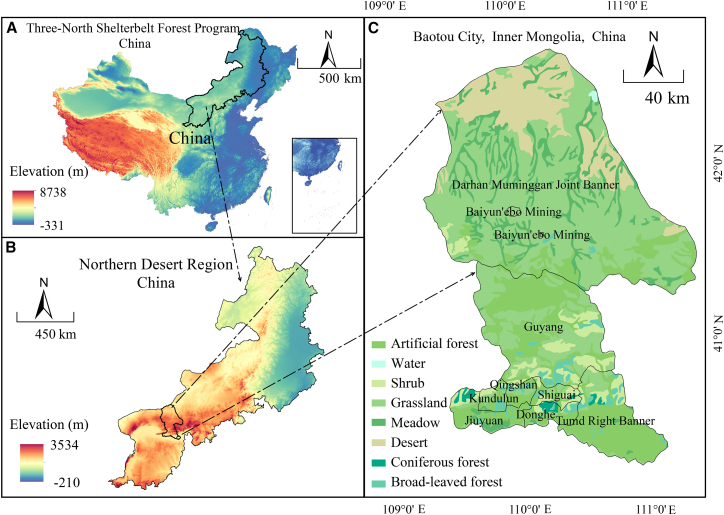
Geographic location and topographic overview of the study area in Baotou City, Inner Mongolia (A) Location of the Three North Shelterbelt Forest Program in China. (B) Study area in the Northern Desert Region. (C) Overview of the study area in Baotou City, Inner Mongolia, China. The study area spans 109°15′–110°26′E and 40°15′–42°43′N, covering approximately 27,768 km^2^. Elevation ranges from 988 to 2,324 m, with a mean elevation of 1,067 m.

This study was conducted within the shelterbelt system in Baotou City, Inner Mongolia, China. The location of the study area^79^ is shown in Figure 12. Influenced by the Yinshan Mountains, the region generally exhibits a topographic pattern of higher elevations in the north and lower elevations in the south, which can be summarized as three major geomorphic gradients: the northern piedmont plateau grasslands, the central mountainous zone, and the southern plains.^80^ The area is characterized by a temperate semi-arid continental monsoon climate,^81^ with an annual precipitation of approximately 300–400 mm, whereas potential evapotranspiration is substantially higher. Together with abundant sunshine averaging 2,900–3,300 h per year and a substantial annual temperature range of 35.5 °C,^82^ this results in a typical environmental combination of low precipitation, high evaporative demand, and strong radiation. Such a climatic background intensifies the constraints of water limitation and atmospheric drought on stomatal regulation, water-use efficiency, and net photosynthetic rate, thereby making interspecific and functional-group differences in photosynthetic acclimation strategies and carbon sequestration capacity among shelterbelt tree species easier to detect and quantify. To characterize key environmental drivers, atmospheric variables were monitored synchronously in spring, summer, and autumn. The seasonal mean values were: PAR averaged 1,044 μmol/m/s, VPD was 3 kPa, relative humidity of air (RHin) was 39%, and carbon dioxide content in air (CO_2_in) was 394 ppm. The study area lies within the core zone for sand control and desertification mitigation of the Three North Shelterbelt Program. Its northern section lies within the Mid-Western Section Northern of Yin Muntains Ecological Comprehensive Management Zone, while the southern part belongs to the Hetao Plain-Yellow River Ecological Comprehensive Management Zone. Our findings can inform carbon sequestration oriented species selection and the precision configuration of shelterbelts in the program area, supporting a strategic shift from large-scale afforestation toward function oriented and precision based design.

**Figure 13:**
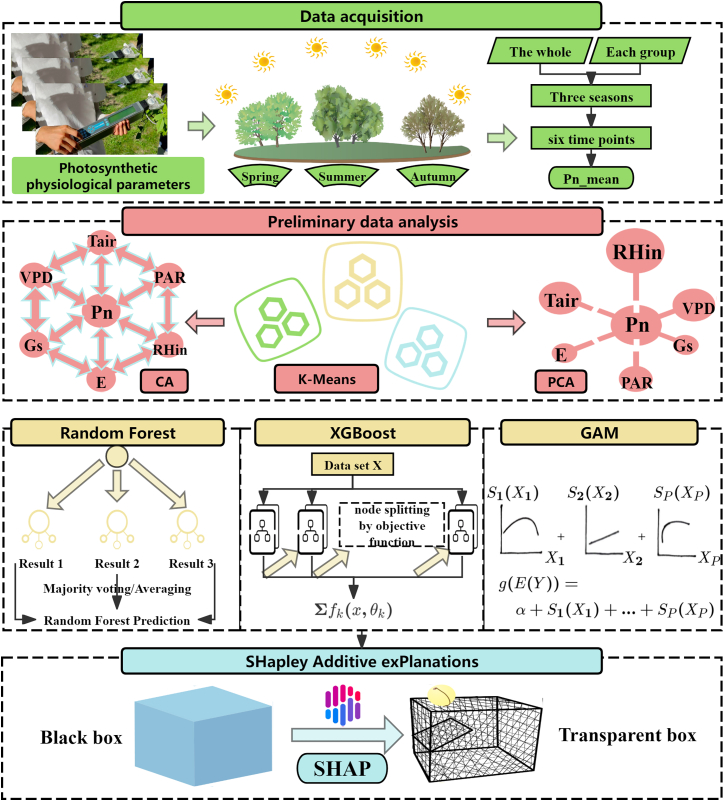
Overall technical route of the study

This study employed a progressive analytical framework integrating functional ecology and explainable machine learning to systematically decipher the drivers of carbon sequestration capacity in Baotou’s shelterbelt species. The overall technical route is depicted in Figure 13, encompassing five core stages: (1) synchronous acquisition and calculation of multi-season photosynthetic physiological and environmental data; (2) identification of carbon sequestration functional groups based on key photosynthetic traits; (3) traditional statistical analysis and dimensionality reduction of environmental factors; (4) comparison and selection of optimal machine learning models for accurate prediction of Pn; (5) application of SHAP explainable analysis to quantitatively uncover key environmental drivers and their modes of action on carbon sequestration capacity across different functional groups.

Field investigations and measurements were conducted in Baotou City, Inner Mongolia, across the growing season of 2024, specifically from April 25 to May 10, July 15 to August 5, and September 10 to October 5. The study targeted 22 typical plant species, comprising 12 tree species and 10 shrub species.

To ensure ecological representativeness, a stratified random sampling strategy was adopted. This involved first stratifying the study area based on dominant tree species and their distribution ranges, followed by the random establishment of standard plots within each stratum, yielding a total of 66 plots. Standard plot sizes were set at 20 × 20 m for trees and 5 × 5 m for shrubs. This methodology was designed to prioritize ecological representativeness over mere geographical coverage. Data collection encompassed basic plot information, leaf area measurements, and photosynthetic parameters. Within each plot, key biometric indices were recorded for each species, including average plant height, mean diameter at breast height for trees, and density. Detailed information on the studied plant species is provided in Table 2.

### Method details

#### Photosynthetic measurements

Measurements of Pn and other indicators were conducted using a CI-340 portable photosynthesis system on windless or breezy sunny days. The CI-340 was calibrated prior to field measurements. After data export, the dataset was inspected and no obvious outliers or missing values were detected; therefore, no additional outlier removal was applied.

Under natural light, representative healthy individuals were selected. For each individual, three sun-exposed mature leaves of similar size were chosen, and three replicate readings were recorded per leaf without detachment. The average of the nine readings was used as the observation at each time point. From 08:00 to 18:00, measurements were performed every 2 h to establish six time points per day, while the order of measurement across species was held uniform across all sampling time points. Physiological and environmental variables were recorded synchronously, for specific measured and derived variable information, refer to Table 3.

#### Calculation of physiological indices

The daily net assimilation amount of the test plants was calculated by integrating the area under the diurnal photosynthetic rate curve, a method standard in plant ecophysiology for obtaining daily carbon fixation from instantaneous measurements.^83^ The calculation formula is:

(Equation 1) $$P_{n}=\sum_{i=1}^{j}\frac{P_{i+1}+P_{i}}{2}\times \left(t_{i+1}-t_{i}\right)\times \frac{3600}{1000}$$

*P*_*n*_ is the total net assimilation per unit leaf area on the measurement day (mmol/m^2^/d). *P*_*i*_ is the instantaneous photosynthetic rate at the initial measurement point (μmol/m^2^/s). *P*_*i*+1_ is the instantaneous photosynthetic rate at the measurement point (μmol/m^2^/s). *t*_*i*_ is the instantaneous time of the initial measurement point (h). *t*_*i*+1_ is the time of the i+1 measurement point (h). *j* is the number of tests.

To ensure a consistent daily timescale in the denominator, transpiration was also aggregated to the daily scale to obtain E using the same trapezoidal integration procedure. Daily WUE was then calculated as:

(Equation 2) $$WUE=\frac{P_{n}}{E}$$

*WUE* is the determination of water use efficiency per unit leaf area per day (μmol/mmol). *E* is the transpiration rate per unit leaf area per day (μmol/m^2^/d).

Because field observations in Baotou showed that Pn at 14:00 was generally higher than that at 12:00, we used Pn at 14:00 to represent the midday level and computed the midday reduction index as:

(Equation 3) $$Midday\;reduction\;index=\frac{P_{n\;\max}-P_{n\;noon}}{P_{n\;\max}}$$

*Midday reduction index* is the Midday reduction index per unit leaf area per day. *P*_*nmax*_ is the maximum net photosynthetic rate per leaf area on the determination day (μmol/m^2^/s). *P*_*nnoon*_ is used to measure the midday net photosynthetic rate per unit leaf area (μmol/m^2^/s).

### Quantification and statistical analysis

#### Functional group classification

To identify species-level functional groups of photosynthetic carbon sequestration, K-means clustering was conducted using functional traits such as Pn, Pn max, and midday reduction index. The optimal number of clusters was determined as k = 4 by jointly considering the Elbow Method and the Silhouette Coefficient. All species were classified into four distinct functional groups: Group 1 with low photosynthesis and strong photoinhibition, Group 2 with high photosynthesis and moderate photoinhibition, Group 3 with high photosynthesis and photoinhibition tolerance, and Group 4 with moderate photosynthesis and photoinhibition tolerance. This functional grouping served as the foundation for all subsequent between and within group comparative analyses.

#### Correlation analysis and dimensionality reduction

Spearman’s rank correlation was used to evaluate the direction and strength of associations between environmental variables (e.g., PAR, Tair, VPD) and Pn. Given the common multicollinearity among environmental predictors, PCA was employed to extract dominant environmental gradients. Prior to PCA, all data were standardized to have a mean of 0 and a standard deviation of 1 to eliminate scale effects.

#### Machine learning modeling, validation, and metrics

To transcend the limitations of traditional linear models and more accurately quantify the nonlinear effects of environmental factors on Pn, this study systematically compared three advanced machine learning regression algorithms: RF, XGBoost, and GAM. Pn was used as the response variable and synchronously measured environmental factors were used as predictors. The dataset was randomly split into training and testing sets with a 7:3 ratio. Key hyperparameters of each model, such as mtry for RF, learning rate and max depth for XGBoost, and smoothing degrees of freedom for GAM, were optimized via 10-fold cross-validation. Model performance was evaluated on the test set using R^2^, RMSE, and MAE, and the optimal model was selected by considering higher R^2^ and lower RMSE/MAE. XGBoost was identified as the best-performing model.

#### Model interpretation

After identifying the optimal machine learning model, which was XGBoost in this study, SHAP analysis was introduced to quantitatively interpret the contribution and directional effect of each environmental factor on the predicted Pn. SHAP analysis was independently conducted on the overall dataset and the four functional group subsets, yielding SHAP values for each environmental factor per observation, which quantitatively reflect its marginal contribution to the model output relative to a baseline, enabling quantitative and attributable interpretation.

#### Data processing and quality control

The CI-340 was calibrated before measurement. After exporting data, we inspected the dataset and found no obvious outliers or missing values; therefore, no additional outlier filtering was applied. If occasional missing values were encountered in specific analyses, a complete-case strategy was used for the corresponding step. All data processing, analysis and partial visualization were accomplished in the R environment, mainly using packages such as tidyverse, caret, xgboost, mgcv, kernelshap and ggplot2.
